# The Association between Cardiac Arrest and Mortality in Patients with Acute Myocardial Infarction Complicated by Cardiogenic Shock

**DOI:** 10.31083/j.rcm2508274

**Published:** 2024-08-01

**Authors:** Qian-feng Xiao, Xin Wei, Si Wang, Ying Xu, Yan Yang, Fang-yang Huang, Mao Chen

**Affiliations:** ^1^Department of Cardiology, West China Hospital, Sichuan University, 610041 Chengdu, Sichuan, China

**Keywords:** cardiac arrest, cardiogenic shock, acute myocardial infarction, mortality

## Abstract

**Background:**

The impact of cardiac arrest (CA) at admission on the prognosis of patients with acute myocardial infarction (AMI) complicated by cardiogenic shock (CS) remains a subject of debate.

**Methods:**

We conducted a retrospective study at West China Hospital from 2018 to 2021, enrolling 247 patients with AMI complicated by CS (AMI-CS). Patients were categorized into CA and non-CA groups based on their admission status. Univariate and multivariate Cox regression analyses were performed, with 30-day and 1-year mortality as the primary endpoints. Kaplan–Meier plots were constructed, and concordance (C)-indices of the Global Registry of Acute Coronary Event (GRACE) score, Intra-aortic Balloon Pump in Cardiogenic Shock (IABP-SHOCK) II score, and IABP-SHOCK II score with CA were calculated.

**Results:**

Among the enrolled patients, 39 experienced CA and received cardiopulmonary resuscitation at admission. The 30-day and 1-year mortality rates were 40.9% and 47.0%, respectively. Neither univariate nor multivariate Cox regression analyses identified CA as a significant risk factor for 30-day and 1-year mortality. In C-statistics, the GRACE score exhibited a moderate effect (C-indices were 0.69 and 0.67, respectively), while the IABP-SHOCK II score had a better predictive performance (C-indices were 0.79 and 0.76, respectively) for the 30-day and 1-year mortality. Furthermore, CA did not enhance the predictive value of the IABP-SHOCK II score for 30-day (*p* = 0.864) and 1-year mortality (*p* = 0.888).

**Conclusions:**

Cardiac arrest at admission did not influence the survival of patients with AMI-CS. Active resuscitation should be prioritized for patients with AMI-CS, regardless of the presence of cardiac arrest.

## 1. Introduction

Acute myocardial infarction (AMI) complicated by cardiogenic shock (CS) presents a formidable challenge in clinical practice, possessing a mortality rate of approximately 40–50% [1]. Notably, AMI patients with cardiac arrest (CA) are more prone to developing CS. In the subset of AMI complicated by CS (AMI-CS), the incidence of CA ranges from 28.5 to 51% [2, 3, 4, 5], and CA is frequently inferred as the primary cause of death in cases of AMI complicated by CS [6].

While previous studies have established CA as a significant risk factor for mortality in patients with acute coronary syndrome (ACS) [7, 8, 9, 10], this association exhibits inconsistency within AMI-CS patients [2, 3, 11]. Some studies suggest that CA may not independently influence all-cause in-hospital or long-term mortality among patients hospitalized with AMI-CS [2, 3]. Notably, these studies reported low proportions of coronary angiography (61.3%) and revascularization (53.3%), potentially compromising the survival probability of CA patients [2, 3]. In contrast, a comprehensive registry study demonstrated higher rates of coronary angiography (88.8%) and revascularization (77.2%) in AMI-CS patients [12].

Recent advancements in acute percutaneous coronary intervention (PCI) and circulatory support devices have significantly improved the short-term prognosis of AMI patients, particularly through early revascularization [1]. Consequently, in the context of active revascularization, the association between CA and the prognosis of patients with AMI-CS has become a subject of debate. In this study, we aimed to assess the impact of CA on AMI-CS outcomes by analyzing data collected from September 2018 to November 2021. Notably, the study cohort exhibited high proportions of coronary angiography (89.5%) and revascularization (80.2%), providing valuable insights into the contemporary landscape of AMI-CS management.

## 2. Methods

### 2.1 Study Population and Definitions

This retrospective study involved 247 patients diagnosed with AMI-CS treated at the West China Hospital of Sichuan University between September 2018 and November 2021. The inclusion criteria encompassed patients within the Society for Cardiovascular Angiography and Interventions (SCAI) shock stages B to E [12, 13]. Cardiac shock was defined as a primary cardiac disorder leading to hypotension (systolic blood pressure <90 mmHg, or vasopressors required to achieve a systolic blood pressure ≥90 mmHg) persisting for over 30 minutes, accompanied by signs of organ hypoperfusion (e.g., altered mental status, oliguria, cold and clammy skin, increased arterial lactate above 2 mmol/L) in a state of normovolemia or hypervolemia [11]. The cardiogenic shock severity was classified according to the SCAI shock stage [12, 13]. Stage B marked the initial phase of CS, identifying patients with clinical evidence of hemodynamic instability (relative hypotension or tachycardia) without hypoperfusion. Stage C denoted classic CS, where patients exhibited hypoperfusion requiring one intervention (pharmacological or mechanical), and lactate was ≥2 mmol/L. Stage D represented deteriorating CS, akin to category C but worsening, with failure of the initial support strategy evident through deteriorating hemodynamics or rising lactate. Stage E indicates the end stage of CS, characterized by actual or impending circulatory collapse. AMI was diagnosed according to the fourth universal definition of myocardial infarction [14]. Criteria included the detection of cardiac troponin values rising and/or falling, with at least one value surpassing the 99th percentile upper reference limit, and the simultaneous presence of symptoms of acute myocardial ischemia, new ischemic ECG changes, development of pathological Q waves, imaging evidence of new myocardial damage, or identification of a coronary thrombus. Cardiac arrest was defined as asystole, pulseless electrical activity, ventricular fibrillation, or pulseless ventricular tachycardia (VT) [3]. CS could still occur due to long-time cardiopulmonary resuscitation (CPR), mechanical complications, and VT storm after AMI, and most continuous CPR patients had no return of spontaneous circulation (ROSC). Hence, the exclusion criteria were (a) the time of CPR was more than 30 min due to cardiac arrest before admission, (b) CS caused by mechanical complications after AMI, (c) CS caused by VT storm, (d) age >90 years, and (e) shock being attributed to other causes, such as septic shock and hemorrhagic shock.

### 2.2 Baseline Data Collection

Demographic characteristics such as body mass index (BMI), blood pressure, heart rate, and respiration status were obtained from nursing records at admission. Medical history (history of hypertension, diabetes, stroke, dyslipidemia, coronary artery disease, peripheral artery disease, and chronic kidney disease), mechanically supported therapies (including circulatory and mechanical respiratory data), and type of AMI were obtained from hospital records. Laboratory parameters were routinely measured after each patient’s admission, and baseline biochemical tests and blood gas results were collected. Coronary artery lesions and intervention information were obtained from in-hospital coronary angiography images and reports. At admission, the left ventricular ejection fraction (LVEF) was evaluated by echocardiography. The Global Registry of Acute Coronary Event (GRACE) and Intra-aortic Balloon Pump in Cardiogenic Shock (IABP-SHOCK) II scores were calculated according to the baseline data of the patients at admission [4, 10]. The GRACE scores of the patients were calculated using the following variables: age, systolic blood pressure, heart rate, serum creatinine level, ST-segment deviation of the electrocardiogram, and cardiac arrest at admission [10]. The IABP-SHOCK Ⅱ scores were calculated by variables such as age, arterial lactate, serum glucose, creatine, history of stroke, and a thrombolysis in myocardial infarction (TIMI) flow grade reaching three after PCI [4].

### 2.3 Study Endpoints/Outcomes

The primary endpoint was death from any cause within 30 days and 1 year. Moreover, we followed the brain injury of patients with CA, while the severity of hypoxic brain damage was evaluated using cerebral performance categories (CPC) [15]. Follow-up information was obtained through telephonic interviews, medical charts, and outpatient visits. Hospital records were used to corroborate all data.

### 2.4 Statistical Analysis

The Kolmogorov–Smirnov test was used to assess data distribution. Continuous variables are presented as the mean or median with each range, and we used independent *t*-tests or the Kruskal–Wallis test to compare variables between groups. Qualitative variables are presented as frequencies and corresponding percentages, and chi-squared or Fisher’s exact tests were used to compare variables between groups. Associations between the clinical variables and endpoints were first assessed using univariate Cox regression analysis. Variables significantly associated with 30-day and 1-year mortality in univariate testing (*p* < 0.10) were further examined using multivariate analysis. Survival rates were presented as Kaplan–Meier plots, and differences between groups were tested using the log-rank test. To investigate the additional predictive value of CA, concordance (C) indices were calculated from the time-to-event data proposed by Harrell *et al*. [16]. Statistical significance was defined as a two-sided *p*-value < 0.05.

Since the mortality rate of AMI-CS was about 40% [1], prior estimates of power were conducted assuming a hazard ratio (HR) of 1.3 for CA; this power analysis showed that a sample size of 247 participants would result in a power of 0.88 (α = 0.05). All analyses were performed using Stata/MP 17.0 (StataCorp LLC, College Station, TX, USA) and R package 4.1.0 (Microsoft, Redmond, WA, USA).

## 3. Results

### 3.1 Baseline Clinical Characteristics between AMI-CS Patients with and without CA

Table 1 summarizes the baseline and clinical characteristics of both the CA and non-CA groups within the cohort of 247 patients experiencing AMI-CS. Among these patients, 39 (15.8%) experienced CA. Most of the cohort was male (74.5%), with no significant difference in sex distribution between the CA and non-CA groups. The average age of all patients was 68 years, with the CA group showing a slightly younger profile (66 ± 13 years vs. 69 ± 13 years) compared to the non-CA group. CA patients exhibited lower mean systolic blood pressure (87 mmHg vs. 92 mmHg, *p* = 0.044) and mean arterial pressure (65.6 mmHg vs. 71.7 mmHg, *p* = 0.002). Additionally, the heart rate was higher in the CA group (100 beats per minute) compared to the non-CA group (96 bpm). Comorbidities, such as hypertension, diabetes mellitus, coronary artery disease (CAD), stroke, peripheral artery disease (PAD), and chronic kidney disease (CKD), were more prevalent in the non-CA group. Baseline lactate (6.9 mmol/L vs. 4.3 mmol/L), glucose (13.34 mmol/L vs. 11.82 mmol/L), and serum creatinine (177.5 µmol/L vs. 144.7 µmol/L) were higher in the CA group. Regarding AMI characteristics, 70.9% of total patients had ST-segment elevation myocardial infarction (STEMI), with 45.3% having anterior STEMI. Patients with CA showed a higher incidence of anterior STEMI (64.1%). Mechanical ventilation was utilized in 75.3% of total patients, with a higher proportion in the CA group (92.3% vs. 72.1%). Mechanical circulatory support was implanted in 42.1% of patients, with a higher proportion in the CA group (59.0% vs. 38.9%). Coronary angiography and revascularization rates were 89.4% and 80.2%, respectively. Additionally, 74.9% of patients had bi- or multivessel lesions, and 83.3% achieved a TIMI flow grade of 3 after PCI. Patients in the non-CA group were more likely to present with multivessel disease (38.2% vs. 48.7%). The GRACE score in the CA group was significantly higher than in the non-CA group (215 vs. 189, *p* = 0.000). According to the IABP-SHOCK II score risk categories, 25.7% and 10.3% of patients were classified as high-risk in the CA and non-CA groups, respectively.

**Table 1.** **Baseline and clinical characteristics of the AMI-related CS patients**.

		Total (n = 247)	CA group (n = 39)	Non-CA group (n = 208)	*p*-value
Demographic data					
	Age, yrs	68 (30–89)	66 (30–89)	69 (32–89)	0.281
	Male	184/247 (74.5)	29/39 (74.4)	155/208 (74.6)	0.983
	BMI, kg/$m^{2}$	23.4 (13.9–35.6)	23.7 (16.8–31.3)	23.4 (13.9–35.6)	0.613
	SBP, mmHg	91 (51–135)	87 (52–122)	92 (51–135)	0.044
	MAP, mmHg	70.7 (40.7–105)	65.6 (40.7–100)	71.7 (41–105)	0.002
	Heart rate, bpm	97 (39–180)	100 (52–180)	96 (39–151)	0.305
Cardiovascular risk factors/CVD (%)					
	Smoking	115/247 (46.6)	20/39 (51.3)	95/208 (45.7)	0.519
	Arterial hypertension	119/247 (48.2)	13/39 (33.3)	106/208 (50.9)	0.043
	Diabetes mellitus	77/247 (31.2)	9/39 (23.1)	68/208 (32.7)	0.234
	History of CAD	48/247 (19.4)	7/39 (17.9)	41/208 (19.7)	0.798
	History of Stroke	19/247 (7.7)	2/39 (5.1)	17/208 (8.2)	0.513
	Dyslipidemia	40/247 (16.3)	3/39 (7.7)	37/208 (17.8)	0.116
	Known PAD	7/247 (2.8)	0/39 (0)	7/208 (3.4)	0.245
	CKD	19/247 (7.7)	2/39 (5.1)	17/214 (8.2)	0.513
Laboratory results					
	Arterial lactate, mmol/L	4.7 (1.0–20)	6.9 (1.1–20)	4.3 (1.0–20)	0.004
	Glucose, mmol/L	12.06 (3.17–37.47)	13.34 (3.17–37.47)	11.82 (3.59–35.91)	0.176
	Serum creatinine, µmol/L	149.9 (51–1014)	177.4 (57–665)	144.7 (51–1014)	0.094
	TnT, ng/L	7295.6 (97.3–10,000)	7346.7 (699–10,000)	7285.9 (97.3–10,000)	0.906
	STEMI (%)	175/247 (70.9)	30/39 (76.9)	145/208 (69.7)	0.363
	Anterior STEMI (%)	112/247 (45.3)	25/39 (64.1)	87/208 (41.8)	0.010
	Ventilation (%)	186/247 (75.3)	36/39 (92.3)	150/208 (72.1)	0.007
	CAG (%)	221/247 (89.5)	34/39 (87.2)	187/208 (89.9)	0.611
Coronary lesions (%)					0.069
	Mono-vessel disease	55/219 (25.1)	12/34 (35.3)	43/185 (23.2)	
	Bi-vessel disease	61/219 (27.9)	9/34 (26.5)	52/185 (28.1)	
	Multi-vessel disease	103/219 (47.0)	13/34 (38.2)	90/185 (48.7)	
	Revascularization (%)	198/247 (80.2)	34/39 (87.2)	164/208 (79.4)	0.231
	Complete revascularization (%)	94/247 (38.1)	18/39 (46.1)	76/208 (36.5)	0.256
	TIMI flow grade 3 after PCI (%)	184/221 (83.3)	29/34 (85.2)	155/187 (82.9)	0.730
	IABP (%)	104/247 (42.1)	23/39 (59.0)	81/208 (38.9)	0.020
	ECMO (%)	6/247 (2.4)	2/39 (5.1)	4/208 (1.9)	0.233
	GRACE score	193 (122–265)	215 (157–265)	189 (122–237)	0.000
IABP-SHOCK II score (%)					0.130
	0–2	137/219 (62.6)	17/35 (48.6)	120/184 (65.2)	
	3–4	54/219 (24.6)	9/35 (25.7)	45/184 (24.5)	
	5–9	28/219 (12.8)	9/35 (25.7)	19/184 (10.3)	
	LVEF, %	40 (15–75)	41 (16–75)	40 (15–70)	0.856

Abbreviations: n, number; AMI, acute myocardial infarction; CS, cardiogenic shock; CA, cardiac arrest; BMI, body mass index; SBP, systolic blood pressure; MAP, mean arterial pressure; bpm, beats per minute; CVD, coronary vascular disease; CAD, coronary artery disease; PAD, peripheral artery disease; CKD, chronic kidney disease; TnT, troponin T; STEMI, ST-segment elevation myocardial infarction; CAG, coronary angiography; TIMI, thrombolysis in myocardial infarction; PCI, percutaneous coronary intervention; IABP, intra-aortic balloon pump; ECMO, extracorporeal membrane oxygenation; GRACE, Global Registry of Acute Coronary Event; IABP-SHOCK, Intra-aortic Balloon Pump in Cardiogenic Shock; LVEF, left ventricular ejection fraction.

### 3.2 The Outcomes between AMI-CS Patients with and without CA

**Fig. 1.**
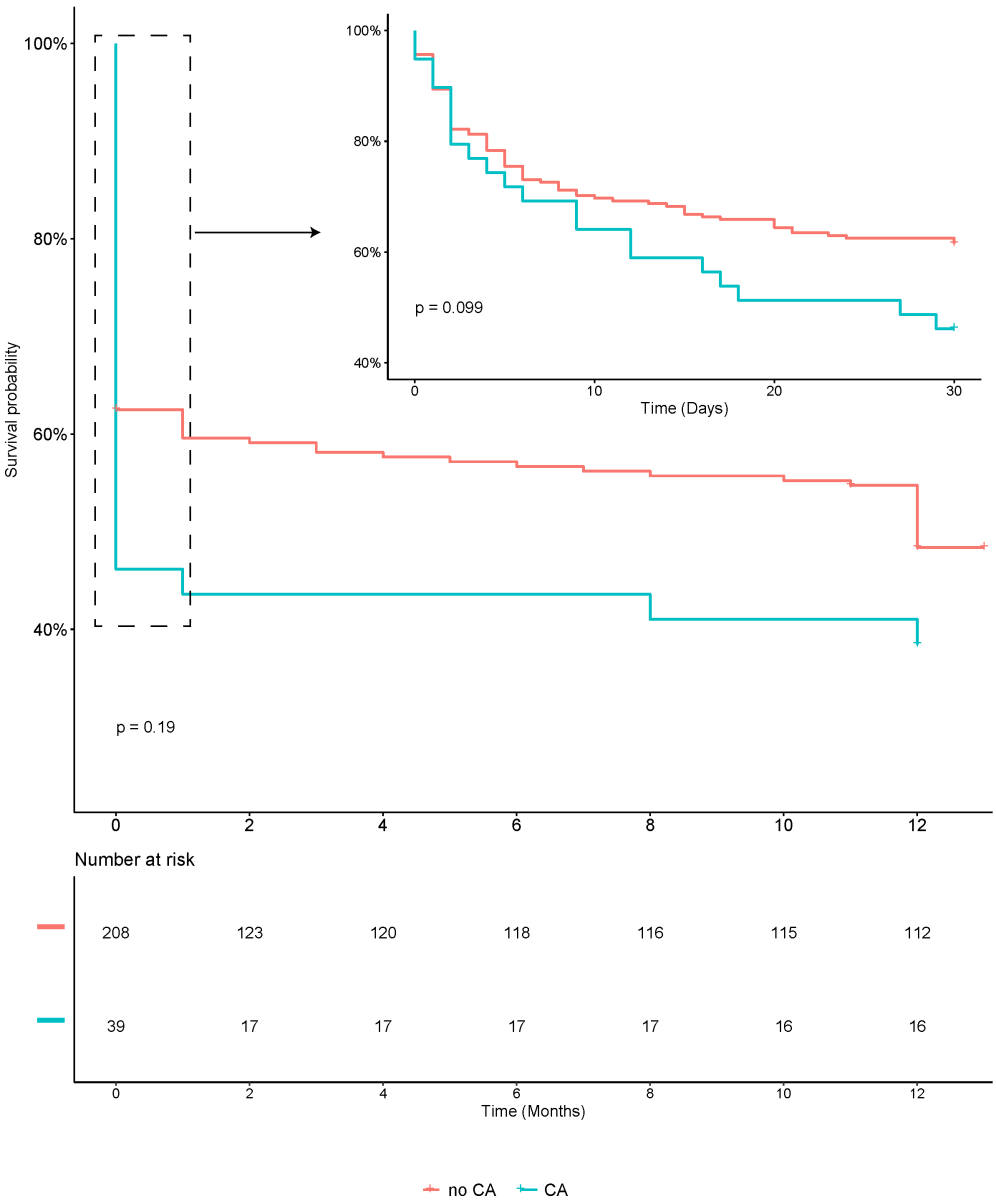
**Kaplan–Meier plot of 30-day and 1-year mortality in AMI-related CS patients with and without CA**. Abbreviations: AMI, acute myocardial infarction; CS, cardiogenic shock; CA, cardiac arrest.

Within the AMI-CS cohort, 101 patients succumbed within 30 days and 116 patients within 1 year, resulting in 30-day and 1-year mortality rates of 40.9% and 47.0%, respectively. In both timeframes, the majority of deaths were attributed to cardiac causes, encompassing sudden cardiac death, ventricular tachycardia or fibrillation, refractory heart failure, or cardiogenic shock, with 93.1% at 30 days and 91.4% at 1 year (Table 2). Survival analysis, as depicted in Fig. 1, did not reveal a significant difference between the groups with and without CA. The overall rate of hypoxic brain damage during the hospital stay was 4.9%. Notably, the CA group exhibited a higher incidence of brain injury (25.6% vs. 1.0%), with 58.3% of these patients succumbing within 30 days and 75% within 1 year. For those surviving cardiac arrest with hypoxic brain injury, the extent of damage was generally mild, as indicated by CPC scores ranging from 1 to 3.

**Table 2.** **Outcomes of AMI-related CS patients**.

		Total	CA group	Non-CA group
Death within 30 days (%)		101/247 (40.9)	21/39 (53.9)	80/208 (38.5)
	Cardiac death	94/101 (93.1)	20/21 (95.2)	74/80 (92.5)
	Non-cardiac death	7/101 (6.9)	1/21 (4.8)	6/80 (7.5)
Death within 1 year (%)		116/247 (47.0)	23/39 (59.0)	93/208 (44.7)
	Cardiac death	106/116 (91.4)	20/23 (87.0)	86/93 (92.5)
	Non-cardiac death	10/116 (8.6)	3/23 (13.0)	7/93 (7.5)
Brain injury (%)		12/247 (4.9)	10/39 (25.6)	2/208 (1.0)
	CPC score (1–3)	3/247 (1.2)	3/39 (7.7)	0/208 (0)
	CPC score (4–5)	9/247 (3.7)	7/39 (17.9)	2/208 (1.0)

Abbreviations: AMI, acute myocardial infarction; CS, cardiogenic shock; CA, cardiac arrest; CPC, cerebral performance category.

### 3.3 Risk Factors for Short- and Long-Term Mortality Caused by AMI in CS Patients

In the univariate Cox regression analysis, CA did not exhibit a significant association with either short- or long-term mortality, with hazard ratios (HRs) of 1.48 (*p* = 0.108) and 1.43 (*p* = 0.127), respectively (Tables 3,4). After adjustment by multivariate analysis, CA continued to lack a significant association with both short- and long-term mortality (HR = 0.88, *p* = 0.669; HR = 1.04, *p* = 0.881, respectively; Tables 3,4 and **Supplementary Table 1**). Exploring the univariate analysis, older age, female gender, higher heart rate, lower SBP and MAP, elevated baseline creatinine, arterial lactate, and glucose levels, presence of hypertension and CKD, TIMI flow grade <3 after PCI, higher GRACE score and IABP-SHOCK II score, lower LVEF, mechanical ventilation, and intra-aortic balloon pump (IABP) implantation were identified as factors associated with increased short- and long-term death (Tables 3,4). Further multivariate analysis revealed that older age, mechanical ventilation, arterial hypertension, higher baseline creatinine and arterial lactate levels, and a TIMI flow grade of <3 after PCI were significant risk factors for both 30-day and 1-year mortality (Tables 3,4).

**Table 3.** **Univariable and multivariable analyses of 30-day mortality for AMI-related CS patients**.

	Univariable analysis of 30-day mortality		Multivariable analysis of 30-day mortality	
	HR (95% CI)	*p*-value	HR (95% CI)	*p*-value
Male	0.51 (0.34–0.77)	0.001		
Age, yrs	1.04 (1.02–1.06)	0.000	1.04 (1.02–1.06)	0.001
BMI, kg/$m^{2}$	0.97 (0.91–1.03)	0.293		
SBP, mmHg	0.98 (0.97–1.00)	0.011		
MAP, mmHg	0.96 (0.95–0.98)	0.003		
Heart rate, bpm	1.01 (1.00–1.02)	0.009		
CA	1.48 (0.92–2.40)	0.108	0.88 (0.48–1.60)	0.669
Ventilation	14.4 (4.58–45.6)	0.000	9.84 (2.38–40.7)	0.002
Arterial hypertension	2.36 (1.57–3.55)	0.000		
Diabetes mellitus	1.38 (0.92–2.06)	0.122		
History of CAD	1.05 (0.65–1.72)	0.835		
History of stroke	1.07 (0.52–2.19)	0.862		
Dyslipidemia	0.98 (0.58–1.68)	0.952		
Known PAD	1.50 (0.55–4.08)	0.427		
CKD	2.28 (1.27–4.08)	0.006		
Arterial lactate, mmol/L	1.15 (1.11–1.19)	0.000	1.15 (1.09–1.20)	0.000
Glucose, mmol/L	1.06 (1.03–1.08)	0.000		
Serum creatinine, µmol/L	3.18 (2.37–4.26)	0.000	2.24 (1.43–3.52)	0.000
TnT, ng/L	1.000 (0.999–1.001)	0.612		
STEMI	0.76 (0.50–1.14)	0.178		
Anterior STEMI	0.98 (0.66–1.45)	0.930		
Revascularization	0.26 (0.17–0.39)	0.000		
Complete revascularization	0.45 (0.29–0.70)	0.000		
TIMI flow grade 3 after PCI	0.17 (0.11–0.28)	0.000	0.20 (0.11–0.31)	0.000
IABP	1.17 (0.79–1.73)	0.428		
ECMO	3.77 (1.65–8.33)	0.002		
LVEF, %	0.96 (0.94–0.98)	0.000		
GRACE score	1.03 (1.02–1.04)	0.000		
IABP-SHOCK II score	1.64 (1.47–1.83)	0.000		

Abbreviations: AMI, acute myocardial infarction; CS, cardiogenic shock; HR, hazard ratio; CI, confidence interval; BMI, body mass index; SBP, systolic blood pressure; MAP, mean arterial pressure; CA, cardiac arrest; CAD, coronary artery disease; PAD, peripheral artery disease; CKD, chronic kidney disease; TnT, troponin T; STEMI, ST-segment elevation myocardial infarction; TIMI, thrombolysis in myocardial infarction; PCI, percutaneous coronary intervention; IABP, intra-aortic balloon pump; ECMO, extracorporeal membrane oxygenation; GRACE, Global Registry of Acute Coronary Event; IABP-SHOCK, Intra-aortic Balloon Pump in Cardiogenic Shock; LVEF, left ventricular ejection fraction.

**Table 4.** **Univariable and multivariable analyses of 1-year mortality for AMI-related CS patients**.

	Univariable analysis of 1 year mortality		Multivariable analysis of 1 year mortality	
	HR (95% CI)	*p*-value	HR (95% CI)	*p*-value
Male	0.55 (0.37–0.81)	0.002		
Age, yrs	1.03 (1.02–1.05)	0.000	1.03 (1.01–1.05)	0.010
BMI, kg/$m^{2}$	0.95 (0.90–1.00)	0.066		
SBP, mmHg	0.99 (0.97–1.00)	0.07		
MAP, mmHg	0.97 (0.96–0.99)	0.001		
Heart rate, bpm	1.01 (1.00–1.02)	0.005		
CA	1.43 (0.90–2.25)	0.127	1.04 (0.59–1.85)	0.881
Ventilation	6.42 (3.13–13.2)	0.000	3.56 (1.60–7.91)	0.002
Arterial hypertension	2.39 (1.64–3.50)	0.000	1.60 (1.01–2.52)	0.045
Diabetes mellitus	1.42 (0.98–2.08)	0.06		
History of CAD	1.05 (0.67–1.66)	0.829		
History of Stroke	1.04 (0.53–2.05)	0.915		
Dyslipidemia	0.95 (0.57–1.57)	0.837		
Known PAD	1.33 (0.49–3.60)	0.579		
CKD	2.01 (1.13–3.59)	0.018		
Baseline arterial lactate, mmol/L	1.15 (1.11–1.19)	0.000	1.12 (1.07–1.18)	0.000
Baseline glucose, mmol/L	1.06 (1.03–1.08)	0.000		
Baseline serum creatinine, µmol/L	2.97 (2.21–4.00)	0.000	2.08 (1.37–3.15)	0.001
TnT, ng/L	1.11 (0.85–1.44)	0.435		
STEMI	0.75 (0.51–1.10)	0.144		
Anterior STEMI	0.91 (0.63–1.32)	0.622		
Revascularization	0.29 (0.19–0.43)	0.000		
Complete revascularization	0.42 (0.28–0.65)	0.000		
TIMI flow grade 3 after PCI	0.21 (0.13–0.33)	0.000	0.23 (0.14–0.37)	0.000
IABP	1.21 (0.84–1.74)	0.317		
ECMO	3.65 (1.60–8.35)	0.002		
LVEF, %	0.96 (0.94–0.98)	0.000		
GRACE score	1.03 (1.02–1.03)	0.000		
IABP-SHOCK II score	1.59 (1.43–1.76)	0.000		

Abbreviations: AMI, acute myocardial infarction; CS, cardiogenic shock; HR, hazard ratio; CI, confidence interval; BMI, body mass index; SBP, systolic blood pressure; MAP, mean arterial pressure; CA, cardiac arrest; CAD, coronary artery disease; PAD, peripheral artery disease; CKD, chronic kidney disease; TnT, troponin T; STEMI, ST-segment elevation myocardial infarction; TIMI, thrombolysis in myocardial infarction; PCI, percutaneous coronary intervention; IABP, intra-aortic balloon pump; ECMO, extracorporeal membrane oxygenation; GRACE, Global Registry of Acute Coronary Event; IABP-SHOCK, Intra-aortic Balloon Pump in Cardiogenic Shock; LVEF, left ventricular ejection fraction.

### 3.4 Predictive Value of CA in Risk Models for Short- and Long-Term Mortality

In the context of C-statistics, the GRACE score exhibited a moderate predictive value for both 30-day and 1-year mortality, with an area under the curve (AUC) of 0.69 [95% confidence interval (CI): 0.64 to 0.75, *p* = 0.000] and 0.67 (95% CI: 0.62 to 0.72, *p* = 0.000), respectively. Conversely, the IABP-SHOCK Ⅱ score demonstrated a high predictive value for both short- and long-term mortality, recording AUC values of 0.79 (95% CI: 0.74 to 0.84, *p* = 0.000) and 0.76 (95% CI: 0.71 to 0.81, *p* = 0.000), respectively. Comparatively, the GRACE score exhibited lower predictive performance than the IABP-SHOCK II score in forecasting short- and long-term mortality among AMI-related CS patients (*p* = 0.003). Additionally, incorporating CA into the IABP-SHOCK II score did not improve the predictive power for either short- or long-term mortality (*p* = 0.863 and 0.888, respectively; Table 5).

**Table 5.** **Models for predicting 30-day and 1-year mortality in C-statistics for AMI-related CS patients**.

	Models to predict 30-day death		Models to predict 1 year death	
	AUC (95% CI)	*p*-value	AUC (95% CI)	*p*-value
GRACE score	0.69 (0.64–0.75)	0.000	0.67 (0.62–0.72)	0.000
IABP-SHOCK II score	0.79 (0.74–0.84)	0.000	0.76 (0.71–0.81)	0.000
IABP-SHOCK II score + CA	0.79 (0.74–0.84)	0.000	0.76 (0.71–0.81)	0.000

Abbreviations: AMI, acute myocardial infarction; CS, cardiogenic shock; AUC, area under the curve; CI, confidence interval; GRACE, Global Registry of Acute Coronary Event; IABP-SHOCK, Intra-aortic Balloon Pump in Cardiogenic Shock; CA, cardiac arrest.

## 4. Discussion

The primary finding of this study was that CA did not emerge as an independent risk factor for either short- or long-term mortality in AMI-CS patients. Despite this, it is noteworthy that CA showed a significant association with the occurrence of brain injury in this patient population. While CA is acknowledged as a crucial element in the GRACE scoring system, our results indicate that the GRACE risk score provides only a moderate predictive value for individuals with AMI-CS. Furthermore, including CA as a risk factor in the IABP-SHOCK II scoring system did not improve its predictive accuracy for either the short- or long-term mortality assessment among AMI-CS patients. This suggests that the predictive value of CA within established risk models may not be as impactful as initially anticipated in shaping the prognosis of individuals with AMI-CS.

Cardiogenic shock after AMI poses a significant threat to life, with an overall 30-day mortality rate of approximately 40–50% [17]. Our study aligns with existing literature, revealing a comparable 30-day mortality rate of 40.7% in patients with AMI-CS. Historically, CA has been identified as a substantial risk factor for mortality in patients with ACS [3, 4, 5, 6]. While the GRACE score, a widely utilized tool for assessing death risk in ACS patients, includes CA as an important predictive factor [18], limited data exists on the correlation between CA and mortality, specifically in AMI-CS patients [11]. Current risk prediction models for CS, such as the IABP-SHOCK Ⅱ score, CARD-SHOCK risk score, SAVE, and ENCOURAGE score, notably omit CA as a risk factor [17]. Our study underscores that CA is not a significant prognostic factor for short- and long-term mortality in AMI-CS patients. Instead, age, lactate level, creatinine level, a TIMI grade <3 after PCI, and ventilation were identified as risk factors for mortality, consistent with previous research [4, 19, 20, 21]. Notably, the GRACE score exhibited moderate predictive performance for mortality in AMI-CS patients, although the addition of CA did not enhance the predictive value of the IABP-SHOCK II score. These observations align with prior studies [2, 3, 5], suggesting that CA may not influence the 7-day, in-hospital, and long-term mortality of AMI-CS patients [2, 3, 5]. The age and comorbidity profile of CA survivors and the simplicity of coronary lesions were proposed to explain these outcomes [4, 19, 20, 21, 22, 23]. Our study also highlighted that effective resuscitation, improved post-resuscitation care, and lower rates of hypoxic brain damage might contribute to the observed low proportion of non-cardiac deaths in the CA group [4, 15, 24]. Contrary to previous reports where hypoxic brain damage and cardiac failure were leading causes of in-hospital death in AMI-CS patients with CA [25], our population predominantly succumbed to cardiac causes. This discrepancy may stem from the impact of effective resuscitation on influencing outcomes, consistent with prior research [26].

This study holds significant clinical implications on multiple fronts. Firstly, it contributes valuable data to the ongoing exploration of whether CA is a prognostic factor for mortality in patients with AMI-CS. This finding emphasizes the importance of proactive rescue efforts for such patients, urging the public to engage in life-saving interventions actively. In China, where awareness regarding cardiac arrest and CPR remains limited [27, 28], our study underscores the critical need for heightened public education on CA and CPR. Importantly, our results suggest that surviving CA does not independently jeopardize the prognosis of AMI-CS patients. Thus, advocating for effective CPR becomes even more imperative, particularly in regions where a substantial number of patients grapple with AMI and cardiogenic shock. Secondly, our study poses a challenge to the GRACE score in predicting outcomes in AMI-CS patients, revealing its limited predictive capability. This observation prompts a reassessment of the GRACE score’s applicability, suggesting that it may not be universally suitable for all patients with ACS. This insight calls for a critical examination of existing risk assessment tools and the potential development of more tailored predictive models for specific subsets of ACS patients, particularly those confronting the complexities of AMI-CS.

While providing valuable insights, this study has limitations. Firstly, the observed rate of CA in our study was 15.8%, lower than previously reported rates [2, 3, 4, 5, 29]. This discrepancy might be attributed to excluding patients who did not survive until hospital admission, potentially leading to an underestimation of the true incidence of CA. The exclusion criteria, particularly limiting CPR time to 30 minutes, aimed to ensure a focus on genuine cases of AMI-CS. Secondly, despite not reaching statistical significance, the 30-day and 1-year mortality rates appeared higher in the CA group (53.9% vs. 38.5% and 59% vs. 44.7%, respectively). While not statistically conclusive, these findings may still bear clinical significance. Previous studies noted comparable outcomes, with one reporting a 62.7% mortality rate at 7 days for the cardiogenic shock population with CA, surpassing that of the non-CA group [3]. Another study, encompassing 1573 patients, identified a relatively higher short-term mortality risk in the CA group, though long-term mortality risk did not reach statistical significance (HR = 1.19, *p* = 0.055) [30]. Indeed, it is essential to acknowledge these trends, even without statistical significance. Thirdly, our study is limited by its retrospective, single-center design, which may limit the generalizability of findings to the broader Chinese population. However, the study population adhered to the criteria established by large prospective randomized controlled trials of AMI-CS [26, 29], and patients spanned various SCAI stages. Fourthly, the lack of exact timing and detailed information on the cardiac arrest events constitutes another limitation. Nevertheless, the meticulous selection of comprehensive clinical data and a robust sample size, pre-determined through careful sample size estimation, mitigate the likelihood of results occurring by chance. Despite these limitations, the study’s findings contribute valuable insights into the complex interplay of cardiac arrest and mortality in the context of AMI-CS.

## 5. Conclusions

In the context of AMI-CS in China, cardiac arrest has emerged as a non-contributory factor in predicting both short- and long-term mortality. Contrary to its significant impact on other cardiac conditions, CA does not independently pose an elevated risk in AMI-CS patients. This finding underscores the importance of active CPR efforts for patients facing AMI-CS, as survival from CA does not inherently compromise their overall prognosis. These results advocate for a proactive and vigorous approach to CPR in this specific patient cohort, emphasizing the need for continued efforts to enhance public awareness and responses to cardiac emergencies in the Chinese population.

## Data Availability

The datasets used and/or analyzed during the current study are available from the corresponding author upon reasonable request.

## Abbreviations

AMI, acute myocardial infarction; CS, cardiogenic shock; AMI-CS, AMI complicated by CS; CA, cardiac arrest; GRACE, Global Registry of Acute Coronary Event; IABP-SHOCK, Intra-aortic Balloon Pump in Cardiogenic Shock; ACS, acute coronary syndrome; PCI, percutaneous coronary intervention; SCAI, cardiovascular angiography, and intervention; VT, ventricular tachycardia; CPR, cardiopulmonary resuscitation; ROSC, return of spontaneous circulation; BMI, body mass index; LVEF, left ventricular ejection fraction; TIMI, thrombolysis in myocardial infarction; CPC, cerebral performance categories; HR, hazard ratio; SBP, systolic blood pressure; MAP, mean arterial pressure; CAD, coronary artery disease; PAD, peripheral artery disease; CKD, chronic kidney disease; STEMI, ST-segment elevation myocardial infarction; IABP, intra-aortic balloon pump; AUC, area under the curve.

## Supplementary Material

Supplementary material associated with this article can be found, in the online version, at https://doi.org/10.31083/j.rcm2508274.
