# Supplementary material for: The Association between Cardiac Arrest and Mortality in Patients with Acute Myocardial Infarction Complicated by Cardiogenic Shock
Source: Rev Cardiovasc Med. 2024 Aug 1;25(8):274. doi: 10.31083/j.rcm2508274 (PMC11366983; doi:10.31083/j.rcm2508274)
Supplement: Supplementary file 1 [file 2153-8174-25-8-274-s1.docx]

Supplementary Table 1. Multivariable analysis for 30-day and 1 year mortality of the AMI related CS patients.

|  | **Multivariable analysis for 30-day mortality** | | **Multivariable analysis in 1 year mortality** | |
| --- | --- | --- | --- | --- |
|  | **HR(95%CI)** | ***p*-Value** | **HR(95%CI)** | ***p*-Value** |
| Age, yrs | 1.04(1.02-1.06) | 0.000 | 1.03(1.01-1.05) | 0.003 |
| CA | 1.56(0.88-2.76) | 0.130 | 1.63(0.94-2.80) | 0.079 |
| Ventilation | 9.44(2.27-39.2) | 0.002 | 3.43(1.54-7.63) | 0.003 |
| Arterial hypertension |  |  | 1.78(1.12-2.80) | 0.014 |
| Baseline glucose, mmol/L | 1.04(1.01-1.07) | 0.005 | 1.03(1.00-1.06) | 0.038 |
| TIMI grade=3 after PCI | 0.19(0.12-0.32) | 0.000 | 0.23(0.15-0.38) | 0.000 |

Abbreviations: AMI=acute myocardial infarction. CS=cardiogenic shock. HR=hazard ratio. CI= confidence interval. CA=cardiac arrest. TIMI= Thrombolysis in Myocardial Infarction. PCI=percutaneous coronary intervention.
